# Age-dependent diminution of female prognostic advantage in gastrointestinal stromal tumors: a retrospective cohort analysis

**DOI:** 10.3389/fimmu.2025.1617019

**Published:** 2025-11-10

**Authors:** Yue Zhang, Qi Liu, Bing Ma, Hao Xu

**Affiliations:** 1 Department of Emergency, The First Hospital of China Medical University, Shenyang, China; 2 Trauma Center, The First Hospital of China Medical University, Shenyang, China; 3 Department of Clinical Epidemiology and Evidence-based Medicine, The First Hospital of China Medical University, Shenyang, China; 4 Department of Gastrointestinal Surgery, The First Hospital of China Medical University, Shenyang, China

**Keywords:** gastrointestinal stromal tumor, SEER, survival, sex-difference, prognosis

## Abstract

**Background:**

Sex and age are significant factors influencing the prognosis of various types of cancer. However, the impact of sex and age on the prognosis of gastrointestinal stromal tumors (GISTs) remains unclear. Investigating the interaction between sex and age may facilitate a more precise assessment of the prognosis of GISTs.

**Methods:**

A retrospective analysis was conducted on a cohort of 5318 patients with GISTs, utilizing the Cox regression model to analyze the disparities in disease-specific survival (DSS) across sex. Subsequently, the cohort after propensity score matching was employed to investigate the prognostic differences attributable to age variations, and restricted cubic spline analysis was utilized to assess the prognostic disparities associated with different sexes and ages in GIST.

**Results:**

This investigation demonstrated substantial sex-based disparities in the clinical characteristics of GIST. With respect to prognosis, males exhibited a significantly elevated hazard ratio (HR) for DSS in comparison to females (HR = 1.40, p<0.001), which persisted following multivariate (HR = 1.38, p=0.006) and propensity score matching analyses (HR = 1.36, p=0.014). Moreover, a significant interaction between age and sex was observed in predicting DSS, notably indicating that younger female subjects (≤50 years) demonstrated a more favorable prognosis relative to their male counterparts.

**Conclusions:**

Female patients with GIST exhibit a more favorable prognosis than males, with this advantage decreasing with advancing age.

## Introduction

Gastrointestinal stromal tumors (GISTs) predominantly occur in elderly individuals and demonstrate similar prevalence across sexes (1–3). Most GISTs exhibit genetic mutations, predominantly activating mutations in KIT and PDGFRA, which function as crucial targets for tyrosine kinase inhibitors (TKIs) in adjuvant therapy (4–6). While the prognosis and risk classification of GIST remain contentious, tumor size, tumor site, and mitotic index are considered critical factors in risk classification (7, 8). However, with the advancement of precision therapy for GISTs, other prognostic factors such as gastrointestinal bleeding, Ki-67, and age > 50 years have attracted the attention of researchers (9–11).

The etiology and progression of numerous cancers are influenced by sex-specific biological disparities, including variations in chromosomal composition and hormonal profiles (12). These disparities are associated with differences in cancer incidence and survival rates. Sex-based differences are correlated with human behavior, attitudes, and social and cultural expectations of the same. Although controversial, GISTs exhibit sex-specific differences (13). Males are predominant in most cancers (14). Studies have reported the discovery of estrogen receptors in GIST (15, 16); however, research examining the disparities in estrogen levels between females and males and their influence on tumor prognosis remains limited. Similarly, age is a significant prognostic factor (10). Emerging evidence suggests that sex hormones, genetics, and the immune system play specific roles in regulating the risk and progression of other malignant tumors (17, 18); however, this has not been conclusively demonstrated in GIST.

The incidence of GIST is marginally higher in males compared to females (19). Moreover, male patients with GIST exhibit more aggressive characteristics, including larger tumor size, higher mitotic index, and increased likelihood of progression to tumor rupture and metastasis. A study confirmed that the prognosis of gist was significantly correlated with sex (20). Another study revealed that patients with GIST aged ≤60 years have a more favorable prognosis compared to those aged >60 years, suggesting that age may serve as an independent prognostic factor for GIST (21). In a multicenter cohort study, the combination of age and sex demonstrated a more favorable prognosis for GIST in patients under 50 years of age and in females (10). However, further validation using large-scale cohort studies is necessary.

Age and sex, as potential prognostic factors, have yet to be incorporated into the existing prognostic risk classification system. This study aimed to assess the prognostic disparities between male and female patients with GISTs through comprehensive cohort analyses, accounting for potential confounding variables that may influence prognosis. This study not only provides a better assessment of the prognosis for patients with GIST but also serves as a reference for the intensity of adjuvant therapy.

## Methods

### Data sources

The research cohort data were derived from the SEER Research Data (2000–2018), 18 Registries, and November 2020 Sub database. A total of 9,957 patients’ information and follow-up data were collected. The criteria for patient inclusion were the availability of data based on the International Classification of Diseases for Oncology, third edition (ICD-O-3), and disease-specific survival (DSS) for individuals diagnosed with GISTs. Screening requirements for potentially eligible patients with GIST included a histologic type of ICD-O-38936 from one of the specified sites, including the Appendix, Colon, Esophagus, Peritoneum, Rectum, Small intestine, and stomach. As the SEER is a publicly available database with anonymized data, this study was exempted by the Ethics Committee of China Medical University.

### Study population

**Figure 1:**
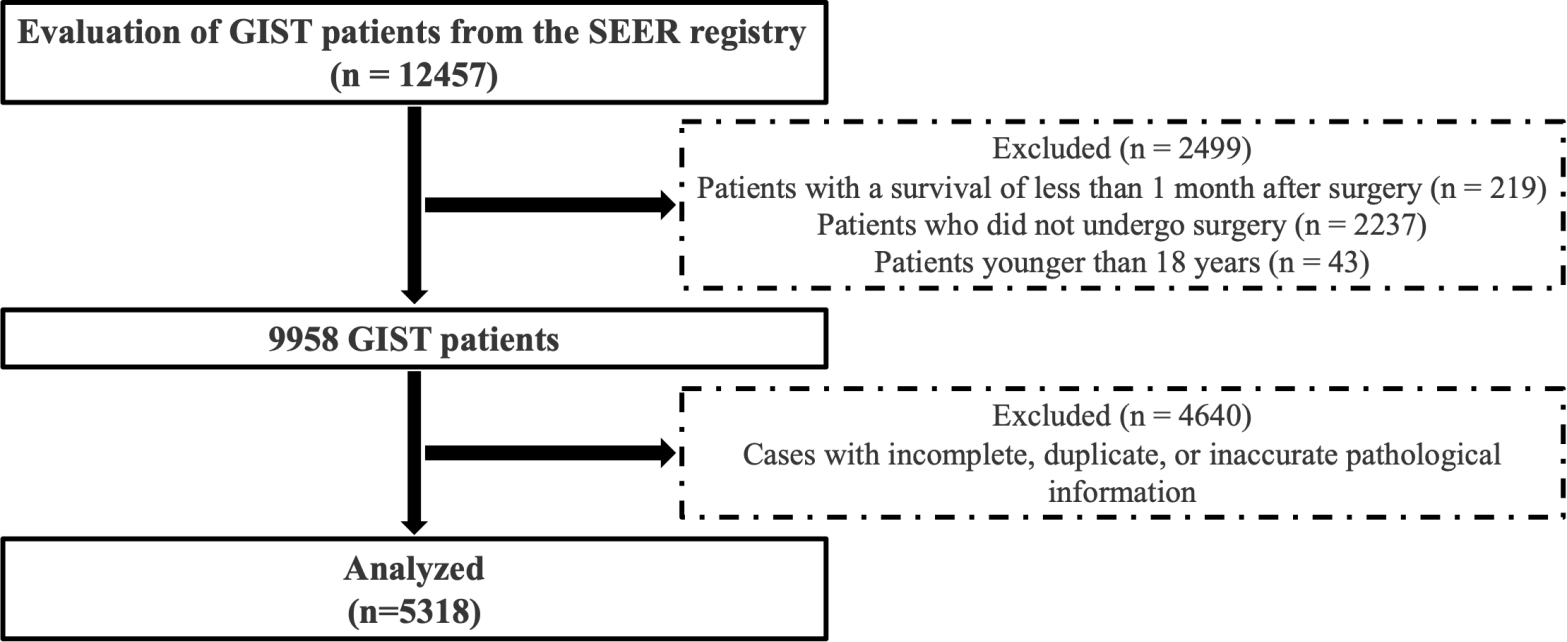
Cohort selection from surveillance, epidemiology, and end results database.

Patients with GIST obtained from the SEER database were excluded based on the following criteria: (a) patients who did not undergo surgical intervention; (b) patients under 18 years of age; (c) patients with a survival duration of less than one month post-surgery; and (d) patients with incomplete, duplicate, or inaccurate pathological data. A total of 5318 cases were included in the statistical analysis. The cohort selection process is illustrated in **Figure 1**.

We excluded patients with missing, duplicate, or inconsistent pathological data. The variables with missing values included tumor size, mitotic index, AJCC stage and tumor site. Other variables such as age, sex, and marital status were complete. Given the lack of a robust multiple imputation strategy for the SEER database in this context and to maintain consistency in the analysis, we opted for complete-case analysis. We acknowledge that this approach may introduce selection bias, particularly if the missing data are not random. However, sensitivity analyses excluding patients with survival <1 month were conducted to assess the robustness of our findings.

### Statistical analysis

Comparisons of baseline characteristics between the sexes were conducted utilizing t-tests and chi-square tests. Survival rates were ascertained using the Kaplan-Meier method, and survival curves were graphically represented. Univariate analysis was performed using the log-rank test, whereas multivariate analysis was performed utilizing the Cox test, with all baseline factors incorporated as adjustment variables. To account for mortality as a competing event, we employed the Fine and Gray subdistribution hazard model in sensitivity analyses to assess the relationship between baseline characteristics and GIST-specific mortality.

Given the potential disparities in variables between the sexes at baseline, propensity score matching (PSM) was employed to address the imbalance. Nearest-neighbor matching with a ratio of 1:1 and a caliper of 0.1 was implemented between different sex groups. The adjusted factors included age, tumor size, marital status, race, origin, tumor site, T stage, N stage, M stage, AJCC stage, radiation, and mitotic index. A standardized mean difference (SMD) of less than 0.1 was considered indicative of balance between variables.

A multiplicative interaction model was employed to analyze the influence of age and sex on the outcomes of patients with GIST. We performed a stratified analysis based on tumor location (gastric vs. non-gastric) to further investigate potential interaction effects between age and sex. To ensure the robustness of the results, a sensitivity analysis was conducted by excluding patients with a survival time of less than one month. To assess the potential non-linear relationship between age and DSS and to visualize how the HR changes continuously with age for sex, we employed restricted cubic spline (RCS) regression. This method is advantageous as it does not impose a pre-specified linear or parametric form on the relationship, allowing the data to reveal the true functional form. The number and positions of the knots, which define the smoothness and flexibility of the spline curve, were chosen based on established statistical recommendations to balance model flexibility and prevent overfitting. The relationships between age, sex, and DSS were investigated utilizing an RCS model. The patients were stratified into three age groups (< 50 years, 50–60 years, and > 60 years), and the association between sex and DSS was examined. To evaluate whether the mediating effect could elucidate the survival differences between females and males, Causal mediation analysis was performed using the ‘mediation’ package in R to quantify the direct and indirect effects of sex on GIST-specific survival. This method is grounded within the counterfactual framework for causal mediation analysis. The mediator variables included AJCC stage, tumor site, T stage, N stage, M stage, radiation, tumor size, and mitotic index. Among these, tumor size was treated as a continuous variable, while all others were included as categorical variables. R software (version 4.3.1) was utilized for all statistical analyses, with the primary packages including survival, tidycmprsk, Matchit, RCS, and mediation. Statistical significance was set at p < 0.05.

## Results

### Sex differences in clinicopathological characteristics of GIST

The study encompassed 9,957 patients with GIST from the SEER database, spanning the period from 2000 to 2018. Following the exclusion of non-qualifying cases, 5,318 patients were ultimately included in the analysis, comprising 2,647 females and 2,671 males. **Table 1** summarizes the clinical and pathological characteristics of the overall cohort and sex subgroups. The demographic factors of men and women differed with respect to their race and marital status. Regarding clinical and pathological features, females exhibited smaller tumor sizes compared to males (66.96 mm vs. 77.52 mm, p < 0.001) and a higher proportion of gastric site involvement (66.2% vs. 60.0%, p < 0.001). Additionally, females demonstrated lower T stage and lower rates of distant metastasis compared to males (8.5% vs. 11%, p = 0.003), lower american joint committee on cancer (AJCC) stage, and lower nuclear mitotic rates (76.18% vs. 80.63%, p < 0.001).

**Table 1:** Characteristics of the overall cohort of GIST patients.

Parameters	Total	Female	Male	*P* value
n	5,318	2,647	2,671	
Age, mean ± SD	63.23 ± 13.26	63.23 ± 13.51	63.22 ± 13.01	0.979
Tumorsize, mean ± SD	72.26 ± 72.14	66.96 ± 71.33	77.52 ± 72.56	<0.001
Marital				<0.001
Single	883(16.6%)	459(17.3%)	424(15.9%)	
Married	3125(58.8%)	1334(50.4%)	1791(67.1%)	
Divorce	454(8.5%)	279(10.5%)	175(6.6%)	
Unknown	254(4.8%)	107(4.0%)	147(5.5%)	
Windowed	557(10.5%)	440(16.6%)	117(4.4%)	
Separated	45(0.8%)	28(1.1%)	17(0.6%)	
Race				0.24
White	3576(67.2%)	1743(65.8%)	1833(68.6%)	
Black	942(17.7%)	480(18.1%)	462(17.3%)	
Asian or Pacific Islander	727(13.7%)	384(14.5%)	343(12.8%)	
American Indian/Alaska Native	15(0.3%)	8(0.3%)	7(0.3%)	
Unknown	58(1.1%)	32(1.2%)	26(1.0%)	
Origin				0.003
Non-Spanish-Hispanic-Latino	4679(88.0%)	2293(86.6%)	2386(89.3%)	
Spanish-Hispanic-Latino	639(12.0%)	354(13.4%)	285(10.7%)	
Race.and.origin				0.003
Hispanic (All Races)	639(12.0%)	354(13.4%)	285(10.7%)	
Non-Hispanic American Indian/Alaska Native	13(0.2%)	8(0.3%)	5(0.2%)	
Non-Hispanic Asian or Pacific Islander	721(13.6%)	380(14.4%)	341(12.8%)	
Non-Hispanic Black	927(17.4%)	469(17.7%)	458(17.1%)	
Non-Hispanic Unknown Race	36(0.7%)	19(0.7%)	17(0.6%)	
Non-Hispanic White	2982(56.1%)	1417(53.5%)	1565(58.6%)	
Tumor site				<0.001
Stomach	3356(63.1%)	1753(66.2%)	1603(60.0%)	
Non-stomach	1962(36.9%)	894(33.8%)	1068(40.0%)	
T stage				<0.001
1	766(14.4%)	434(16.4%)	332(12.4%)	
2	1849(34.8%)	965(36.5%)	884(33.1%)	
3	1565(29.4%)	757(28.6%)	808(30.3%)	
4	1138(21.4%)	491(18.5%)	647(24.2%)	
N stage				0.206
0	5167(97.2%)	2580(97.5%)	2587(96.9%)	
1	151(2.8%)	67(2.5%)	84(3.1%)	
M stage				0.003
0	4798(90.2%)	2421(91.5%)	2377(89.0%)	
1	520(9.8%)	226(8.5%)	294(11.0%)	
AJCC stage				<0.001
1	2844(53.5%)	1529(57.8%)	1315(49.2%)	
2	905(17.0%)	436(16.5%)	469(17.6%)	
3	952(17.9%)	417(15.8%)	535(20.0%)	
4	617(11.6%)	265(10.0%)	352(13.2%)	
Radiation				0.209
Yes	30(0.6%)	11(0.4%)	19(0.7%)	
No	5288(99.4%)	2636(99.6%)	2652(99.3%)	
Mitotic index				<0.001
≤5/50HPF	2996(78.39%)	1468(76.18%)	1528(80.63%)	
>5/50HPF	826(21.61%)	459(23.82%)	367(19.37%)	

### Male as an independent risk factor for GIST prognosis

**Figure 2:**
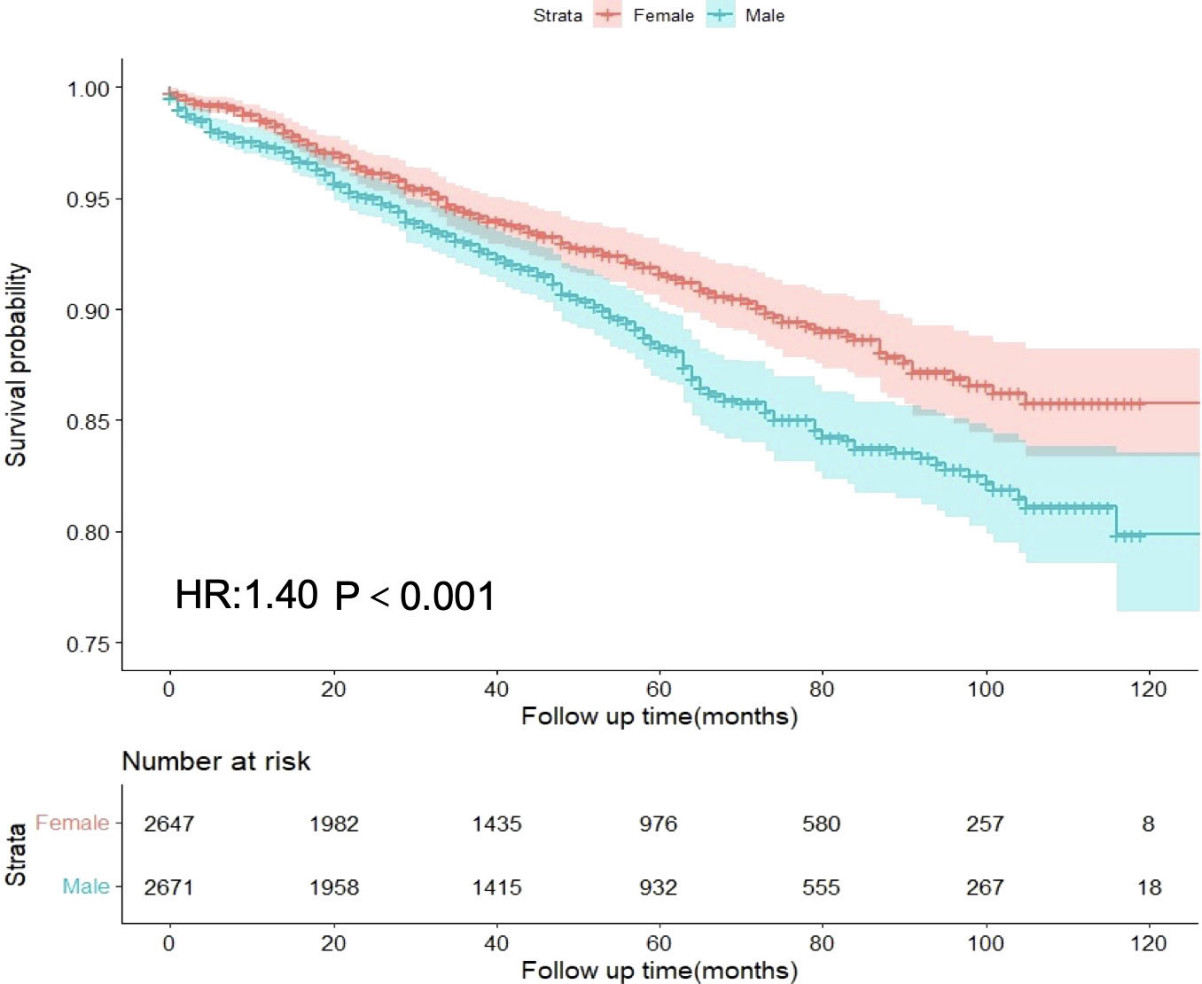
Differences in disease-specific survival between male and female GIST patients in the overall cohort.

In the entire cohort, females had a median follow-up time of 44 months, whereas males had a median follow-up time of 43 months. In the female GIST cohort, 190 patients experienced disease progression (7.18%), whereas in the male cohort, 262 patients experienced disease progression (9.81%). Univariate analysis revealed that males exhibited a higher hazard ratio (HR) for DSS than females (HR = 1.40, 95%CI: 1.16-1.68, p<0.001) (**Figure 2**). Multivariate analysis was adjusted for the following variables: race, origin, tumor site, T stage, N stage, M stage, radiation, age, tumor size, marital status, AJCC stage, and mitotic rate. The results demonstrated that the HR for DSS remained significantly higher in males compared to females (HR = 1.38, 95%CI: 1.10-1.74, p=0.006). The associations between sex and DSS remained robust in the competing risk models (**Supplementary Table S1**).

Following PSM for sex, all variables between the two subgroups exhibited adequate balance (**Supplementary Figure S1**, **Supplementary Table S2**). Prognostic analysis of the PSM-adjusted cohort revealed statistically significant differences in DSS for HR between sexes (HR = 1.36, 95% CI: 1.06-1.73, p=0.014) (**Table 2**). Consequently, both multivariable analysis and PSM models indicated that male sex remained an independent risk factor for GIST.

**Table 2:** Characteristics of the overall cohort of GIST patients before and after PSM.

Parameters	Primary cohort		PSM cohort	
	HR(95%CI)	P-value	HR(95%CI)	P-value
Age	1.04(1.03,1.05)	<0.001	1.04(1.03,1.05)	<0.001
Tumor size	1.00(1.00,1.00)	0.04	1.00(1.00,1.00)	0.218
Gender(Male vs Female)	1.38(1.10,1.74)	0.006	1.36(1.06,1.73)	0.014
Marital				
Single vs Married	0.70(0.52,0.94)	0.018	0.66(0.48,0.90)	0.010
Single vs Divorced	1.15(0.76,1.73)	0.501	1.08(0.70,1.68)	0.728
Single vs Unknown	0.90(0.52,1.53)	0.686	0.79(0.43,1.46)	0.457
Single vs Widowed	0.88(0.57,1.35)	0.551	0.88(0.53,1.46)	0.616
Single vs Separated	1.03(0.32,3.29)	0.970	0.83(0.20,3.43)	0.793
Race				
White vs Black	2.05(0.28,15.18)	0.483	–	–
White vs Asian or Pacific Islander	–		–	
White vs American Indian/Alaska Native	4.02(1.26,12.81)	0.019	3.63(0.87,15.14)	0.077
White vs unknown	–		–	–
Origin	1.19(0.83,1.71)	0.335	1.38(0.93,2.03)	0.108
Race and origin				
Hispanic vs Non-Hispanic American Indian/Alaska Native				
Hispanic vs Non-Hispanic Asian or Pacific Islander				
Hispanic vs Non-Hispanic Black	0.74(0.10,5.58)	0.770	–	–
Hispanic vs Non-Hispanic Unknown Race				
Hispanic vs Non-Hispanic Non-Hispanic White				
Tumor site	1.10(0.87,1.40)	0.406	1.14(0.88,1.48)	0.330
T Stage				
T2 vs T1	0.42(0.25,0.70)	0.001	0.35(0.20,0.63)	<0.001
T3 vs T1	0.75(0.45,1.25)	0.270	0.62(0.35,1.09)	0.097
T4 vs T1	0.80(0.45,1.42)	0.434	0.60(0.32,1.13)	0.115
N Stage	1.73(1.04,2.94)	0.042	1.58(0.88,2.84)	0.127
M Stage	2.00(0.93,4.32)	0.077	2.35(0.89,6.21)	0.086
AJCC				
Stage II vs Stage I	1.88(1.21,2.92)	0.005	2.44(1.49,4.00)	<0.001
Stage III vs Stage I	3.73(2.24,6.21)	<0.001	4.77(2.67,8.52)	<0.001
Stage IV vs Stage I	3.66(1.50,8.89)	0.004	4.44(1.50,13.12)	0.007
Radiation	0.43(0.19,0.98)	0.043	0.35(0.14,0.85)	0.02
Mitotic index	1.34(1.01,1.77)	0.046	1.17(0.85,1.61)	0.324

### Sex-age interaction in GIST prognosis

**Figure 3:**
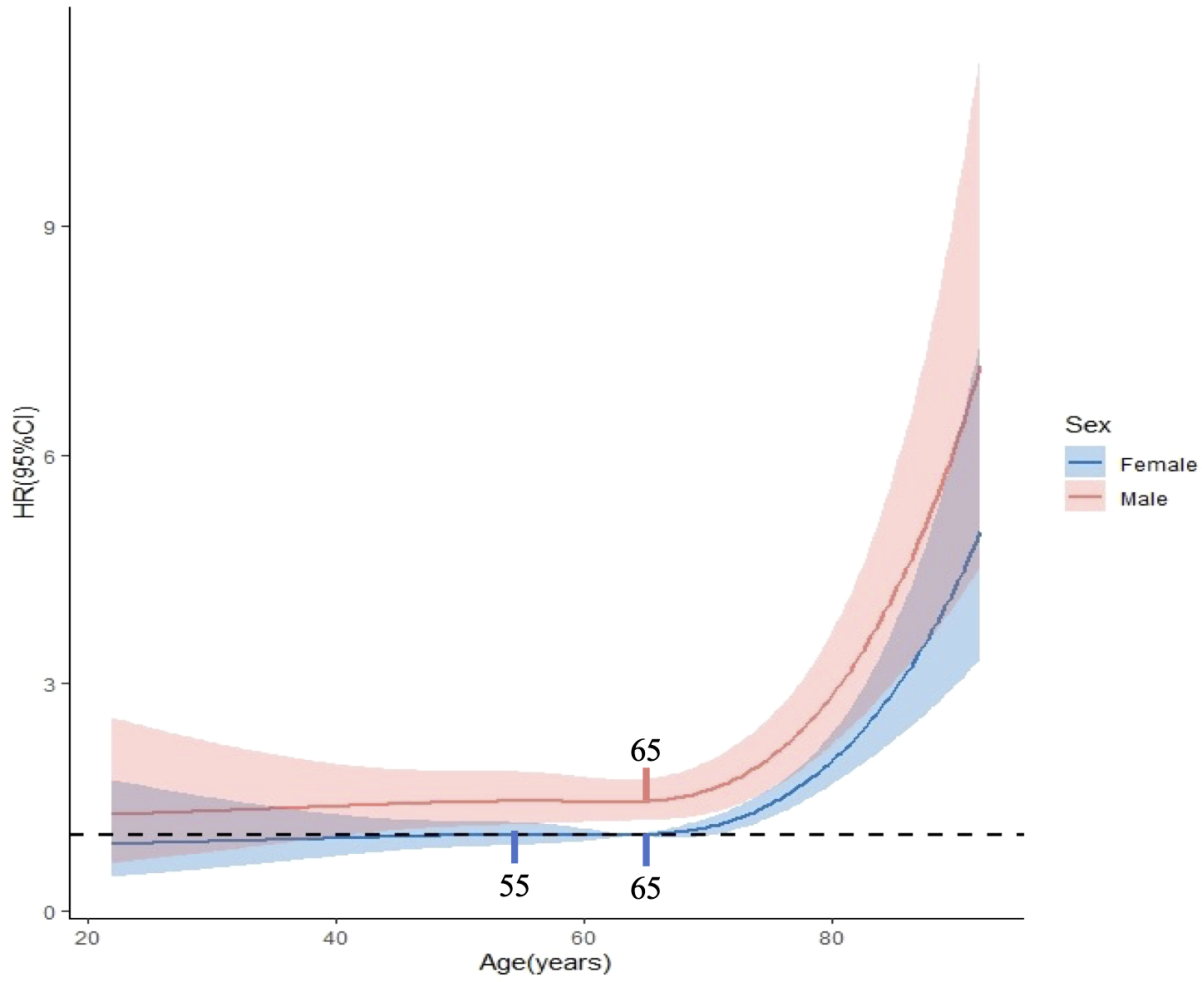
Interaction between age and gender in prognostic prediction of GISTs. The critical age was identified as 65 years for males and at both 55 and 65 years for females.

A statistically significant interaction was observed between age and sex in predicting the prognosis of GIST (p = 0.025). In the gastric tumor cohort, no significant interaction was observed between age and sex (P = 0.23). In contrast, a statistically significant interaction between age and sex was identified in the non-gastric tumor cohort (P = 0.027). Sensitivity analysis after excluding patients with a survival time of less than one month confirmed that the interaction between age and sex remained statistically significant in predicting GIST-specific survival (P = 0.029). RCS analysis revealed that the HR for DSS in males gradually increased with age, demonstrating a significant elevation after 65 years (p < 0.001 for non-linearity). Conversely, the HR for DSS in females displayed a gradual increase between the ages of 20 and 55, maintained relative stability between 55 and 65 years, and subsequently increased rapidly after 65 years (p = 0.010 for non-linearity). Moreover, the HR for DSS in males consistently exceeded that in females (**Figure 3**).

### Age attenuates sex prognosis disparities in GIST

**Figure 4:**
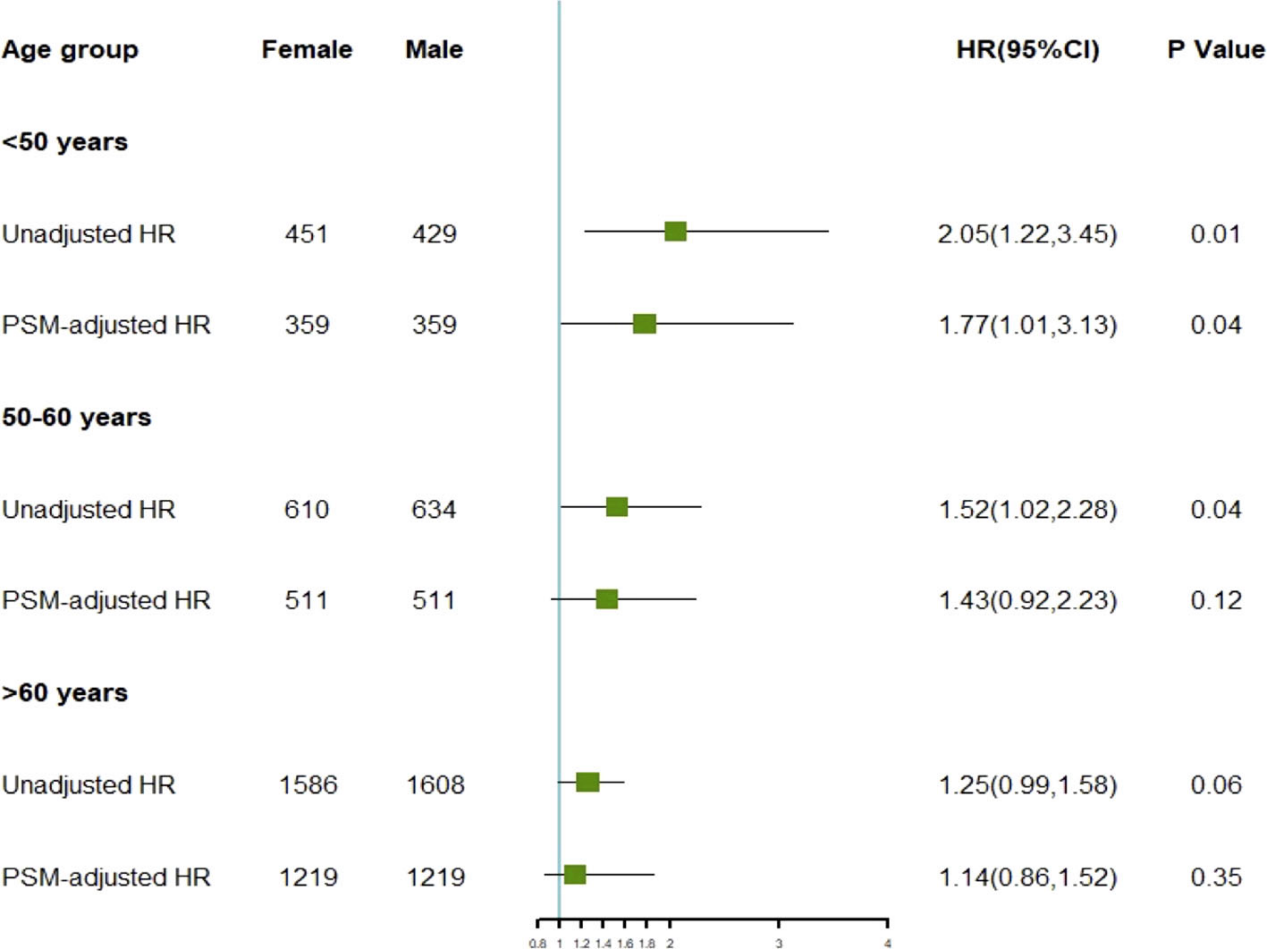
HR of disease-specific survival for different genders before and after adjustment across various age subgroups.

**Figure 5:**
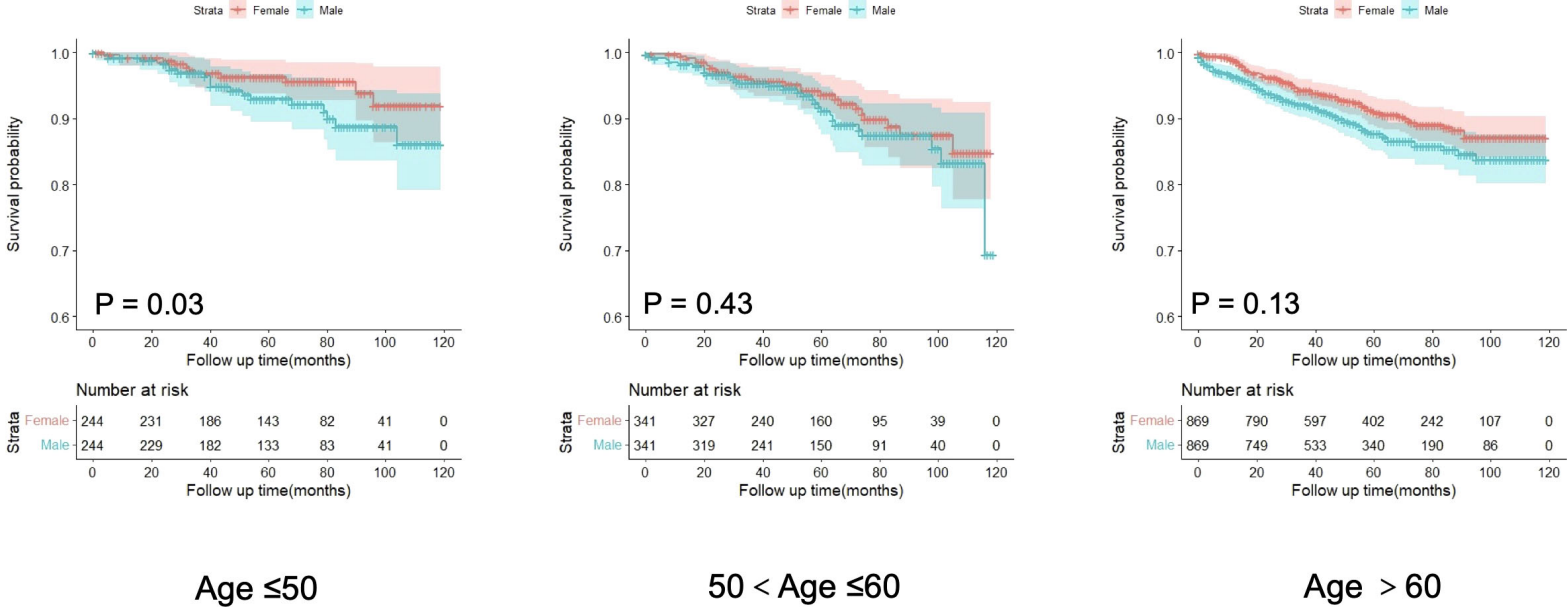
Analysis of the differences in disease-specific survival of GISTs among age subgroups after propensity score matching.

To investigate the differences in prognosis between sexes across different age subgroups, we categorized age into three subgroups (≤ 50 years, 51–60 years, and > 60 years) and conducted an analysis, as described in **Table 3**. Through subgroup analysis, we identified variations in the pathological parameters between males and females across different age groups. To validate these potential differences, we performed PSM to adjust for baseline characteristics between the different sexes within each age subgroup. After PSM, the intergroup variables within each age subgroup were well-balanced (**Supplementary Figure S2**). The results of the subgroup analysis indicated that the differences in DSS between sexes diminished with increasing age, demonstrating statistical significance only in the ≤ 50 years age subgroup. No significant differences were observed between the other age groups (**Figure 4**). In these three subgroups, survival analysis after PSM revealed that the prognostic differences between males and females were present only in the ≤ 50 years subgroup, where the HR for DSS in males was higher than that in females, with no such differences observed in the other two subgroups (**Figure 5**). This suggests that after adjusting for various factors, younger and middle-aged female patients with GIST exhibit a favorable prognosis; however, this advantage diminishes with increasing age.

**Table 3:** Differences in GIST prognosis by gender and age subgroups.

Characteristics	≤50 years			51-60 years			>60 years		
Total No.	Female (n=451)	Male (n=429)	*P* value	Female (n=610)	Male (n=634)	*P* value	Female (n=1586)	Male (n=1608)	*P* value
Age (mean ± SD)	41.86 ± 7.02	42.17 ± 7.07	0.511	55.88 ± 2.85	56.06 ± 2.82	0.247	72.14 ± 7.58	71.66 ± 7.40	0.072
Tumor size (mean ± SD)	65.42 ± 69.47	82.89 (68.80)	<0.001	72.51 ± 83.16	84.00 ± 78.86	0.013	65.26 ± 66.72	73.53 ± 70.69	0.001
Marital			0.02			<0.001			<0.001
Single	140(31.0%)	120(28.0%)		120(19.7%)	122(19.2%)		199(12.5%)	182(11.3%)	
Married	238(52.8%)	264(61.5%)		347(56.9%)	406(64.0%)		749(47.2%)	1121(69.7%)	
Divorce	39(8.6%)	16(3.7%)		90(14.8%)	48(7.6%)		150(9.5%)	111(6.9%)	
Unknown	25(5.5%)	24(5.6%)		26(4.3%)	44(6.9%)		56(3.5%)	79(4.9%)	
Windowed	4(0.9%)	3(0.7%)		19(3.1%)	5(0.8%)		417(26.3%)	109(6.8%)	
Separated	5(1.1%)	2(0.5%)		8(1.3%)	9(1.4%)		15(0.9%)	6(0.4%)	
Race			0.322			0.355			0.006
White	291(64.5%)	282(65.7%)		408(66.9%)	425(67.0%)		1044(65.8%)	1126(70.0%)	
Black	91(20.2%)	73(17.0%)		121(19.8%)	120(18.9%)		268(16.9%)	269(16.7%)	
Asian or Pacific Islander	57(12.6%)	68(15.9%)		72(11.8%)	78(12.3%)		255(16.1%)	197(12.3%)	
American Indian/Alaska Native	3(0.7%)	1(0.2%)		1(0.2%)	6(0.9%)		4(0.3%)	0(0.0%)	
Unknown	9(2.0%)	5(1.2%)		8(1.3%)	5(0.8%)		15(0.9%)	16(1.0%)	
Origin			1			0.089			0.005
Non-Spanish-Hispanic-Latino	366(81.2%)	349(81.4%)		521(85.4%)	563(88.8%)		1406(88.7%)	1474(91.7%)	
Spanish-Hispanic-Latino	85(18.8%)	80(18.6%)		89(14.6%)	71(11.2%)		180(11.3%)	134(8.3%)	
Race and origin			0.399			0.308			<0.001
Hispanic (All Races)	85(18.8%)	80(18.6%)		89(14.6%)	71(11.2%)		180(11.3%)	134(8.3%)	
Non-Hispanic American Indian/Alaska Native	3(0.7%)	1(0.2%)		1(0.2%)	4(0.6%)		4(0.3%)	0(0.0%)	
Non-Hispanic Asian or Pacific Islander	56(12.4%)	68(15.9%)		72(11.8%)	77(12.1%)		252(15.9%)	196(12.2%)	
Non-Hispanic Black	90(20.0%)	72(16.8%)		118(19.3%)	119(18.8%)		261(16.5%)	267(16.6%)	
Non-Hispanic Unknown Race	5(1.1%)	2(0.5%)		4(0.7%)	2(0.3%)		10(0.6%)	13(0.8%)	
Non-Hispanic White	212(47.0%)	206(48.0%)		326(53.4%)	361(56.9%)		879(55.4%)	998(62.1%)	
Tumor site			0.002			0.001			0.028
Stomach	266(59.0%)	207(48.3%)		399(65.4%)	353(55.7%)		1088(68.6%)	1043(64.9%)	
Non-stomach	185(41.0%)	222(51.7%)		211(34.6%)	281(44.3%)		498(31.4%)	565(35.1%)	
T stage			<0.001			0.002			0.001
1	76(16.9%)	46(10.7%)		122(20.0%)	79(12.5%)		236(14.9%)	207(12.9%)	
2	156(34.6%)	121(28.2%)		185(30.3%)	205(32.3%)		624(39.3%)	558(34.7%)	
3	140(31.0%)	142(33.1%)		170(27.9%)	177(27.9%)		447(28.2%)	489(30.4%)	
4	79(17.5%)	120(28.0%)		133(21.8%)	173(27.3%)		279(17.6%)	354(22.0%)	
N stage			0.126			0.569			0.871
0	438(97.1%)	407(94.9%)		595(97.5%)	614(96.8%)		1547(97.5%)	1566(97.4%)	
1	13(2.9%)	22(5.1%)		15(2.5%)	20(3.2%)		39(2.5%)	42(2.6%)	
M stage			0.104			0.175			0.044
0	416(92.2%)	381(88.8%)		548(89.8%)	553(87.2%)		1457(91.9%)	1443(89.7%)	
1	35(7.8%)	48(11.2%)		62(10.2%)	81(12.8%)		129(8.1%)	165(10.3%)	
AJCC stage			0.003			0.001			0.001
1	244(54.1%)	184(42.9%)		343(56.2%)	290(45.7%)		942(59.4%)	841(52.3%)	
2	86(19.1%)	84(19.6%)		102(16.7%)	111(17.5%)		248(15.6%)	274(17.0%)	
3	77(17.1%)	97(22.6%)		94(15.4%)	139(21.9%)		246(15.5%)	299(18.6%)	
4	44(9.8%)	64(14.9%)		71(11.6%)	94(14.8%)		150(9.5%)	194(12.1%)	
Radiation			1			0.394			0.373
Yes	3(0.7%)	3(0.7%)		1(0.2%)	4(0.6%)		7(0.4%)	12(0.7%)	
No	448(99.3%)	426(99.3%)		609(99.8%)	630(99.4%)		1579(99.6%)	1596(99.3%)	
Mitotic index			0.167			0.014			0.057
≤5/50HPF	254(80.6%)	241(75.8%)		359(81%)	322(73.9%)		915(80.5%)	905(77.2%)	
>5/50HPF	61(19.4%)	77(24.2%)		84(19%)	114(26.1%)		222(19.5%)	268(22.8%)	

### Sex-mediated GIST prognosis via tumor staging and biological behavior

**Figure 6:**
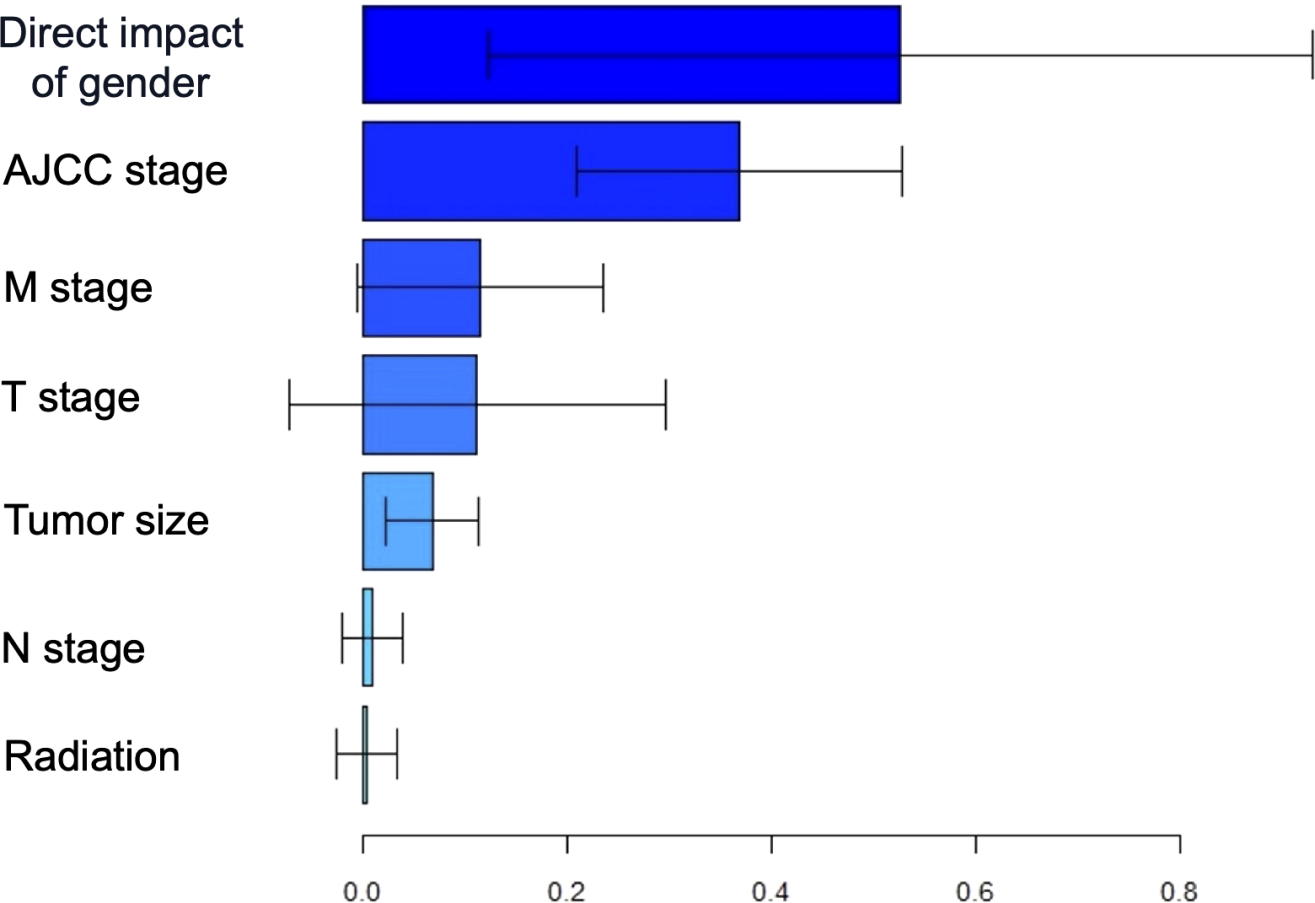
The estimation of direct and indirect effects contributing to the using multiple mediation analyses with gender disparity in DSS.

To further investigate the impact of sex on DSS mediated by other factors, we conducted a multiple mediation analysis (**Figure 6**). Based on the contribution of sex to the mediation of DSS, the five most influential variables, ranked in descending order, were AJCC stage (36.8%), M stage (11.6%), T stage (11.2%), tumor size (6.8%), and N stage (0.9%). These findings suggest that sex may indirectly influence the prognosis of GIST by affecting its biological behavior.

## Discussion

Over the past few decades, the incidence of GIST has increased (22). Currently, the impact of sex on the prognosis of GIST remains a subject of debate. In this extensive retrospective cohort study, our findings indicated that females exhibited an advantage in prognosis. However, this advantage gradually diminished with advancing age. Our results suggest an interaction between sex and age in GIST, potentially conferring a protective effect in middle-aged and younger female patients. These findings may contribute to the development of tailored, personalized treatments for patients with GISTs and potentially improve their prognosis. The predominant methodologies currently employed for the prognostic evaluation of GIST are the NIH or modified NIH risk classification systems. As precision therapy continues to advance, alternative assessment approaches have begun to contribute to the risk stratification of GIST. Numerous studies have substantiated that several factors may contribute to poor prognosis in GIST, including tumor rupture, positive surgical margins, KIT exon 11 mutations, and gastrointestinal hemorrhage (9, 23). Furthermore, studies have indicated that an elevated preoperative Glasgow Prognostic Score may function as a predictive factor for long-term prognosis in patients with GISTs (24). Nevertheless, the combined influence of age and sex on GIST prognosis remains unexplored in the current literature.

Several studies have indicated that elderly patients generally exhibit poorer prognoses than younger patients. Physiological alterations leading to decreased immune function, coupled with relatively poor tolerance and treatment responses, may contribute to this phenomenon. Consequently, when investigating the impact of sex on the prognosis of GIST, age-related factors should be considered. As age increases, the risk of various adverse outcomes significantly increases, resulting in a poorer prognosis for patients with GISTs. The disparities between males and females in GIST, as well as the confounding effects of age and sex on different tumor progressions, have been established in thyroid cancer and nasopharyngeal carcinoma (25, 26).However, these relationships remain unclear in the context of GIST.

Approximately 75% of patients with GIST are diagnosed after the age of 50 (27). This factor significantly influenced the decision to establish an age threshold of 50 years. In the ≤ 50 years age subgroup, females initially demonstrated a more favorable prognosis compared to males. Nevertheless, as age increases, the prognostic disparity between the two sexes progressively diminishes, resulting in a reduction in the initial prognostic advantage observed in females. This phenomenon may be attributed to the decline in immune function and organ dysfunction associated with the aging process. However, younger female patients with GISTs maintain higher levels of estrogen compared to their male counterparts. After the age of 50 years, estrogen levels decrease significantly, consequently leading to older female patients with GISTs losing their prognostic advantage. Furthermore, the decline in estrogen levels following menopause may impair anti-tumor immune responses, which could partly explain why the survival advantage observed in women diminishes with age. Estrogen is known to modulate the function of immune cells, including enhancing the anti-tumor activity of CD8+ T cells (28, 29). Additionally, estrogen receptors are expressed on various immune cells, and estrogen signaling has been shown to suppress M2 tumor-associated macrophages (30). The different effects of male and female sex hormones on cancer immunity may lead to a higher incidence of cancer in men and a poorer prognosis (31, 32). The decline in estrogen levels, coupled with age-related immune dysfunction, may synergistically undermine anti-tumor immunity in older women, thereby diminishing their survival advantage in GIST.

A multicenter study investigated the influence of sex and age on prognosis, demonstrating that female patients with GISTs under 50 years of age exhibit a favorable prognosis (10). This finding is consistent with the results of the present study. However, this investigation solely categorized patients by age and sex without examining the dynamic changes in the HR of DSS with increasing age. A separate study similarly identified advanced age as an independent adverse prognostic factor for patients with GIST, while female sex was considered a protective factor in comparison to male (33). Contemporary research on sex generally indicates that females exhibit a more favorable prognosis than males (34, 35). A favorable prognosis has been observed in female patients aged ≤ 21 years with gastric GIST (36). Collectively, these findings indicate that female patients with GISTs may benefit from the protective effects associated with estrogen levels.

Estrogen plays a crucial role in regulating the female reproductive system and normal physiological functions; however, it may also contribute to certain malignancies. Specifically, estrogen is hypothesized to promote endometrial and breast cancers (37, 38). Conversely, estrogen may exert a protective effect against certain neoplasms. Specifically, estrogen has been observed to reduce the risk of colorectal cancer (39). Consequently, the dual role of estrogen in tumors necessitates further investigation. Estrogen may exert an inhibitory effect on GIST progression, potentially elucidating the improved prognosis observed in female patients. However, additional research is required to substantiate this hypothesis.

Nevertheless, this study has several limitations. Most notably, the SEER database does not include information on KIT and PDGFRA mutation status, nor does it provide records of TKI therapy, which is a critical treatment for GIST and significantly influences patient outcomes. The absence of this information may confound the interpretation of the observed sex-based survival differences. If there are sex-related variations in mutation profiles or responses to TKI treatment, our results may overstate or understate the true effect of sex on survival. Furthermore, the lack of these data complicates mediation analyses aimed at elucidating the indirect pathways through which sex influences survival via tumor stage and biological behavior. Without accounting for mutation status and TKI treatment, the estimated contributions of tumor stage and other factors may be biased, as these factors are themselves influenced by molecular characteristics and the efficacy of TKI therapy. Therefore, although our findings highlight significant prognostic disparities related to sex and age, they require validation in studies incorporating genetic mutation and TKI treatment data.

In conclusion, this study investigated the prognostic disparities among various age groups and sexes within a GIST cohort. Elucidating the interaction between sex and age in GIST prognosis will contribute to a more comprehensive understanding of the biological characteristics of GIST, potentially improving patient treatment outcomes and facilitating the development of more efficacious individualized treatment strategies.

## Data Availability

The original contributions presented in the study are included in the article/**Supplementary Material**. Further inquiries can be directed to the corresponding author.
