# Supplementary figures and images for: Age-dependent diminution of female prognostic advantage in gastrointestinal stromal tumors: a retrospective cohort analysis
Source: Front Immunol. 2025 Nov 10;16:1617019. doi: 10.3389/fimmu.2025.1617019 (PMC12640972; doi:10.3389/fimmu.2025.1617019)

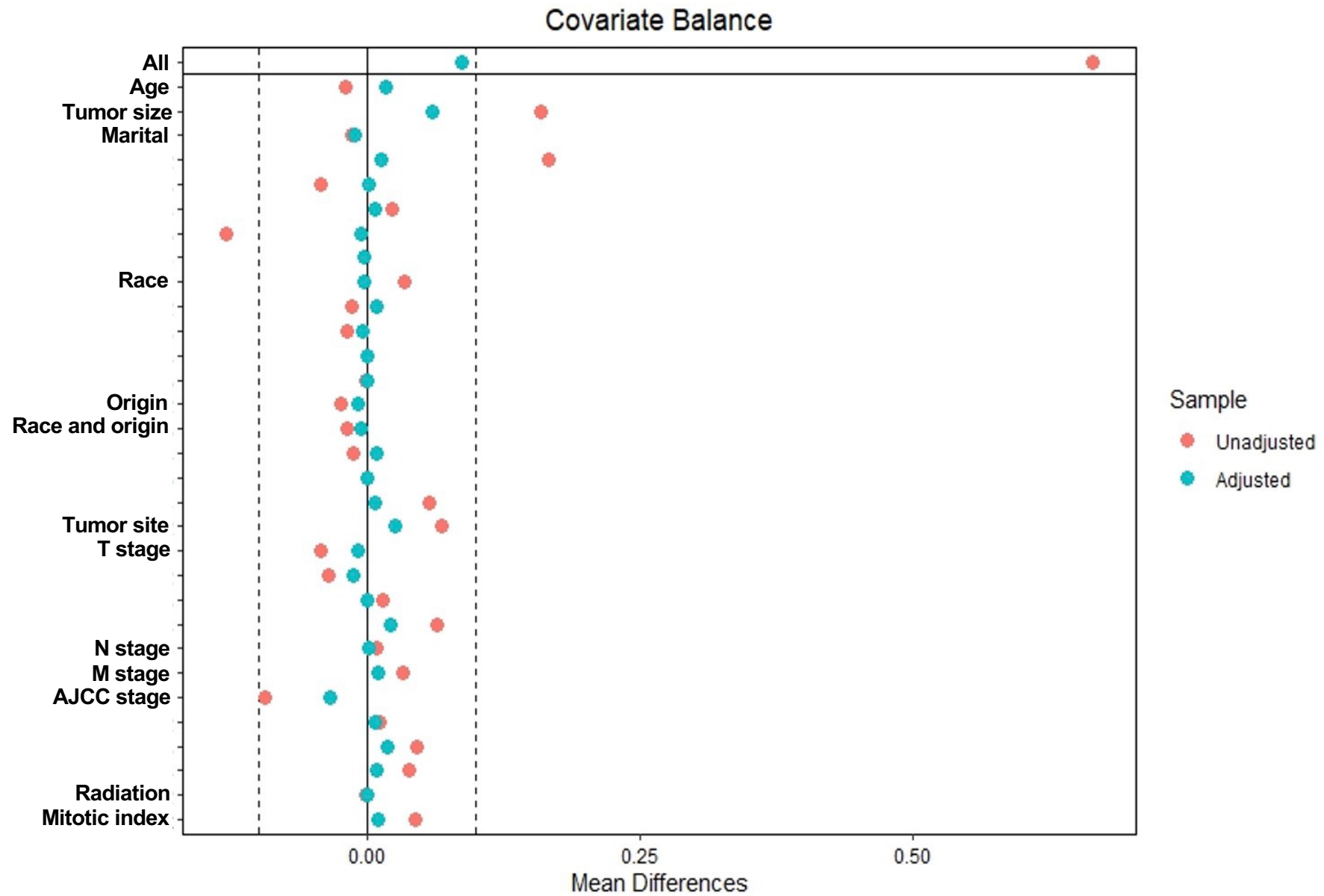

Figure S1. Baseline parameters were balanced after propensity score matching.

Supplement: Supplementary file 1 [file Image1.pdf]

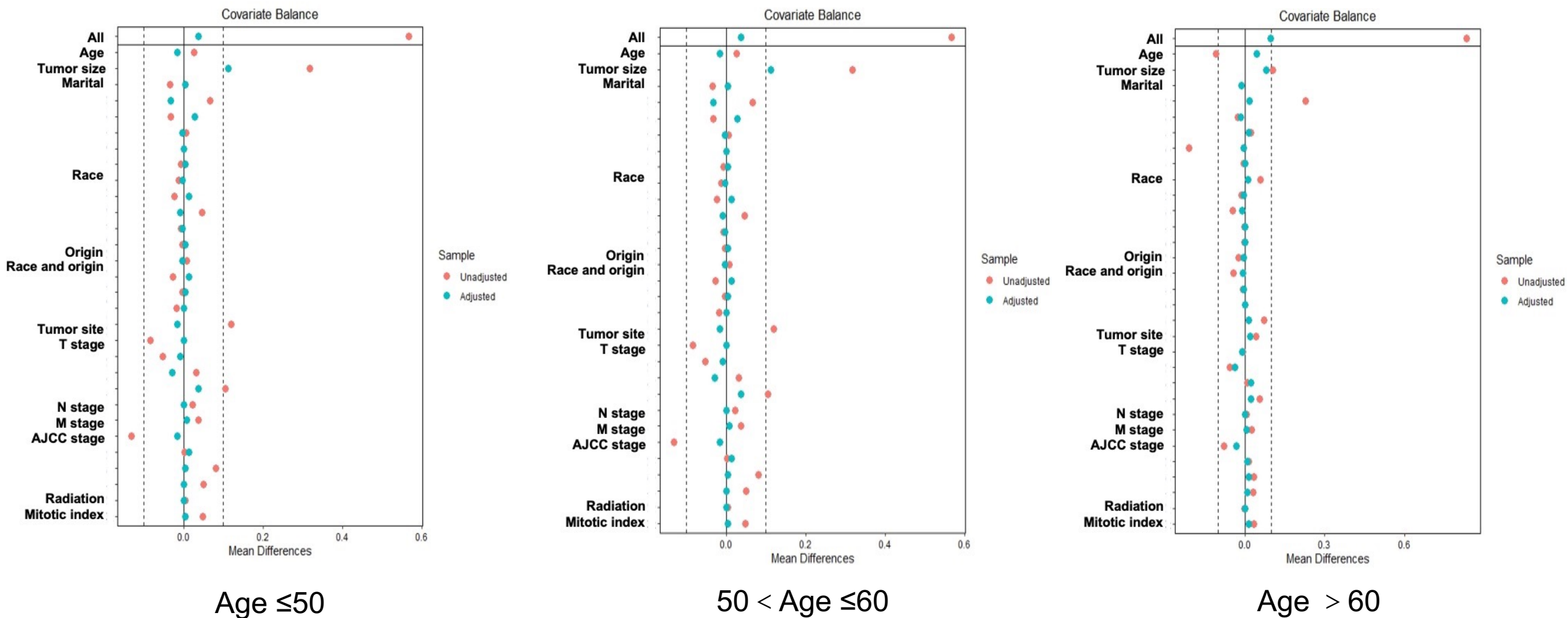

Figure S2 Baseline characteristics of each age subgroup after propensity score matching.

Supplement: Supplementary file 2 [file Image2.pdf]
