# Supplementary material for: Age-dependent diminution of female prognostic advantage in gastrointestinal stromal tumors: a retrospective cohort analysis
Source: Front Immunol. 2025 Nov 10;16:1617019. doi: 10.3389/fimmu.2025.1617019 (PMC12640972; doi:10.3389/fimmu.2025.1617019)
Supplement: Supplementary file 3 [file Table1.docx]

**Table S1 Analysis of the overall cohort of GIST Patients using a competing risk model**

| Parameters | Competing Risk model | |
| --- | --- | --- |
|  | HR（95%CI） | P-value |
| Age | 1.02(1.01,1.03) | <0.001 |
| Tumor size | 1(1.00,1.00) | 0.003 |
| Sex（Male vs Female） | 1.23(1.01,1.50) | 0.041 |
| Marital |  |  |
| Single vs Married | 0.71(0.55,0.92) | 0.01 |
| Single vs Divorced | 1.08(0.75,1.57) | 0.66 |
| Single vs Unknown | 0.80(0.50,1.28) | 0.35 |
| Single vs Widowed | 0.82(0.55,1.20) | 0.3 |
| Single vs Separated | 0.51(0.15,1.71) | 0.27 |
| Race |  |  |
| White vs Black | 1.43(1.11,1.84) | 0.006 |
| White vs Asian or Pacific Islander | 1.09(0.81,1.47) | 0.58 |
| White vs American Indian/Alaska Native | 3.35(1.50,7.49) | 0.58 |
| White vs unknown | - | - |
| Origin | 1.23(0.92,1.64) | 0.16 |
| Tumor site | 1.09(0.89,1.34) | 0.38 |
| T stage |  |  |
| T2 vs T1 | 0.45(0.29,0.70) | <0.001 |
| T3 vs T1 | 0.66(0.42,1.04) | 0.071 |
| T4 vs T1 | 0.70(0.43,1.15) | 0.16 |
| N stage | 1.53(0.93,2.50) | 0.094 |
| M stage | 1.73(0.89,3.35) | 0.11 |
| AJCC stage |  |  |
| Stage II vs Stage I | 2.22(1.48,3.31) | <0.001 |
| Stage III vs Stage I | 5.08(3.47,7.45) | <0.001 |
| Stage IV vs Stage I | 4.81(2.28,10.1) | <0.001 |
| Radiation | 0.39(0.21,0.72) | 0.003 |
| Mitotic index | 2.52(1.92,3.29) | <0.001 |
