# Supplementary material for: Age-dependent diminution of female prognostic advantage in gastrointestinal stromal tumors: a retrospective cohort analysis
Source: Front Immunol. 2025 Nov 10;16:1617019. doi: 10.3389/fimmu.2025.1617019 (PMC12640972; doi:10.3389/fimmu.2025.1617019)
Supplement: Supplementary file 4 [file Table2.docx]

**Table S2 Variation in standardized mean differences pre- and post-propensity score matching**

| **Factor** | **Pre-SMD** | **Post-SMD** |
| --- | --- | --- |
| Age | -0.0204 | 0.0175 |
| Tumor size | 0.1586 | 0.0598 |
| Marital_ Single | -0.0137 | -0.0112 |
| Marital_ Married | 0.1666 | 0.0132 |
| Marital_ Divorced | -0.0434 | 0.0007 |
| Marital_ Unknown | 0.0222 | 0.0066 |
| Marital_ Widowed | -0.1291 | -0.0059 |
| Marital_ Separated | -0.0027 | -0.0033 |
| Race_ White | 0.0347 | -0.0033 |
| Race_ Black | -0.0150 | 0.0079 |
| Race_ Asian or Pacific Islander | -0.0179 | -0.0046 |
| Race_ American Indian/Alaska Native | -0.0006 | 0.0000 |
| Race_ Unkown | -0.0012 | 0.0000 |
| Origin | -0.0250 | -0.0092 |
| Race.and.origin_ Hispanic | -0.0250 | -0.0092 |
| Race.and.origin_ Non-Hispanic American  Indian/Alaska Native | -0.0006 | 0.0000 |
| Race.and.origin_ Non-Hispanic Asian or Pacific Islander | -0.0179 | -0.0053 |
| Race.and.origin_ Non-Hispanic Black | -0.0128 | 0.0079 |
| Race.and.origin_ Non-Hispanic Unknown Race | -0.0001 | 0.0000 |
| Race.and.origin_ Non-Hispanic White | 0.0564 | 0.0066 |
| Tumor site | 0.0684 | 0.0251 |
| T-stage_I | -0.0421 | -0.0092 |
| T-stage_II | -0.0362 | -0.0125 |
| T-stage_III | 0.0143 | 0.0000 |
| T-stage_IV | 0.0640 | 0.0218 |
| N-stage | 0.0080 | 0.0007 |
| M-stage | 0.0326 | 0.0092 |
| AJCC-stage_I | -0.0946 | -0.0343 |
| AJCC-stage_II | 0.0106 | 0.0073 |
| AJCC-stage_III | 0.0454 | 0.0185 |
| AJCC-stage_IV | 0.0386 | 0.0086 |
| Radiation | -0.0010 | 0.0000 |
| Mitotic count | 0.0445 | 0.0106 |

Pre-SMD：Pre-propensity score matching standardized mean differences

Post-SMD：Post-propensity score matching standardized mean differences
